# Inflammatory and Metabolic Biomarkers Associated with In-Hospital Mortality in Patients Hospitalized with Heart Failure

**DOI:** 10.3390/metabo16080534

**Published:** 2026-07-29

**Authors:** Elena Cojocaru, Victorița Șorodoc, Cristian Cojocaru, Laurențiu Șorodoc, Raluca Ecaterina Haliga

**Affiliations:** 1 Grigore T. Popa University of Medicine and Pharmacy, 700115 Iași, Romania; elena.cojocaruu@umfiasi.ro (E.C.); victorita.sorodoc@umfiasi.ro (V.Ș.); laurentiu.sorodoc@umfiasi.ro (L.Ș.); raluca.haliga@umfiasi.ro (R.E.H.); 2 Second Internal Medicine Clinic of the “St. Spiridon” County Emergency Clinical Hospital, 700111 Iași, Romania

**Keywords:** heart failure, inflammatory biomarkers, metabolic biomarkers, C-reactive protein, systemic immune–inflammation index, neutrophil-to-lymphocyte ratio, N-terminal pro-B-type natriuretic peptide, obesity paradox, in-hospital mortality

## Abstract

**Highlights:**

**What are the main findings?**

- Patients presenting with decompensated heart failure had a more pronounced inflammatory profile than those with compensated heart failure.
- Higher CRP and SII levels were associated with in-hospital mortality in adjusted analyses.

**What are the implications of the main findings?**

- Inflammatory and metabolic abnormalities often coexist in hospitalized patients with heart failure.
- Routine inflammatory markers may complement short-term clinical assessment, although their predictive value requires further validation.

**Abstract:**

**Background/Objectives**: Inflammatory and metabolic abnormalities are common in patients hospitalized with heart failure (HF), particularly during episodes of decompensation. Their associations with short-term in-hospital outcomes remain incompletely understood. **Methods:** We retrospectively reviewed 499 patients with documented chronic HF who were admitted for acute medical conditions between January and May 2024. The cohort was not limited to patients admitted for HF exacerbation; patients were classified as having compensated or decompensated HF at admission. Body mass index (BMI) was available for 498 patients, whereas HF status at admission was available for all 499 patients. Clinical, metabolic, and laboratory parameters were analyzed, with particular attention to C-reactive protein (CRP), neutrophil-to-lymphocyte ratio (NLR), platelet-to-lymphocyte ratio (PLR), systemic immune–inflammation index (SII), and N-terminal pro-B-type natriuretic peptide (NT-proBNP). Patients were stratified according to BMI category (<25 kg/m^2^ or ≥25 kg/m^2^) and HF status at admission. Associations with all-cause in-hospital mortality were evaluated using multivariable logistic regression analysis. **Results:** Patients with decompensated HF at admission had higher CRP, NLR, PLR, SII, and NT-proBNP levels, as well as lower hemoglobin and eGFR values. All-cause in-hospital mortality was numerically higher in patients with BMI < 25 kg/m^2^ than in those with BMI ≥ 25 kg/m^2^ (8.4% vs. 4.2%), but the difference was not statistically significant (Pearson’s χ^2^ test, *p* = 0.063). In adjusted analyses, CRP and SII were associated with all-cause in-hospital mortality, whereas NT-proBNP was not. Patients with higher BMI more often had glycemic and lipid abnormalities, whereas those with lower BMI had a more pronounced inflammatory profile. The apparent area under the curve (AUC) values were 0.793 for the CRP-based model and 0.814 for the SII-based model; after bootstrap correction, the optimism-corrected AUC values were 0.729 and 0.754, respectively. **Conclusions:** Higher CRP and SII levels were associated with all-cause in-hospital mortality after adjustment for selected clinical variables.

## 1. Introduction

Patients hospitalized with heart failure (HF) frequently present with renal dysfunction, anemia, metabolic abnormalities, and elevated inflammatory marker levels, particularly during episodes of decompensation [1,2,3]. Several of these abnormalities are more common in patients with more severe disease and in those who die during hospitalization, raising interest in the relationship between systemic inflammation and short-term outcomes in HF [4].

HF is increasingly viewed as a systemic disease rather than an isolated hemodynamic disorder. In addition to cardiac dysfunction, many patients have persistent inflammatory activation together with metabolic and nutritional abnormalities [5]. Chronic inflammation has been associated with endothelial dysfunction, insulin resistance, altered energy metabolism, skeletal muscle loss, and progressive cardiorenal impairment [6,7]. These disturbances may become more pronounced during episodes of acute decompensation [8,9].

Inflammatory marker levels in HF may increase because of congestion, renal dysfunction, ischemia, infection, comorbidities, or the physiological stress accompanying acute decompensation. In hospitalized patients, inflammatory biomarkers therefore likely reflect several overlapping mechanisms rather than a single pathological process [10].

Several metabolic abnormalities observed in HF are also closely related to higher body mass index (BMI). In the general population, higher BMI is associated with increased cardiovascular risk [11]. However, among patients with established HF, higher BMI has not consistently been associated with worse short-term outcomes. Several studies have reported lower mortality among overweight and obese hospitalized patients with HF, an observation commonly referred to as the obesity paradox [12,13,14]. The mechanisms underlying this association remain uncertain.

Among patients with HF, lower BMI is often associated with frailty, malnutrition, reduced muscle mass, or cardiac cachexia [15,16,17]. Such patients may tolerate acute decompensation less well and may therefore have worse short-term outcomes during hospitalization [18]. At the same time, BMI has important limitations because it does not distinguish between adiposity, fluid retention, muscle mass, or nutritional reserve [19,20].

Inflammatory and metabolic biomarkers may provide additional information during the clinical evaluation of hospitalized patients with HF. C-reactive protein (CRP) reflects systemic inflammatory activation, whereas the neutrophil-to-lymphocyte ratio (NLR), platelet-to-lymphocyte ratio (PLR), and systemic immune–inflammation index (SII) are hematologic indices derived from routine blood counts and have been associated with inflammatory burden in several cardiovascular conditions [21,22]. Metabolic parameters such as blood glucose, glycated hemoglobin (HbA1c), lipid profile, albumin, and BMI may also reflect nutritional status, metabolic imbalance, and catabolic changes in HF [23].

Although inflammatory biomarkers and BMI have both been studied in HF populations, few studies have evaluated their joint associations with short-term in-hospital outcomes in heterogeneous hospitalized cohorts [24,25,26,27]. Many previous studies focused either on chronic ambulatory HF populations or on isolated biomarkers without simultaneously evaluating inflammatory profiles, metabolic abnormalities, and acute decompensation [28,29,30,31]. Consequently, the relationships among inflammatory abnormalities, BMI category, and short-term outcomes remain incompletely characterized in real-world populations of hospitalized patients with HF.

The present study evaluated the associations among routinely available inflammatory biomarkers, BMI category, and in-hospital mortality in a single-center cohort of hospitalized patients with a documented history of HF. Metabolic characteristics and N-terminal pro-B-type natriuretic peptide (NT-proBNP) levels were also examined in relation to HF decompensation and adverse short-term outcomes.

## 2. Materials and Methods

### 2.1. Study Design and Population

This retrospective observational study was conducted at the Second Internal Medicine Clinic of the “St. Spiridon” County Emergency Clinical Hospital, Iași, Romania. We reviewed admissions from 1 January to 31 May 2024 and identified adults with documented chronic HF. This period was selected because the medical records were most complete and the variables required for the study were recorded consistently during these months.

The diagnosis of HF was based on the available clinical data, laboratory findings, and electronic medical records, in accordance with current European Society of Cardiology guidelines [32].

Patients aged ≥ 18 years with available clinical and laboratory data at admission were eligible for inclusion. Patients with acute infections, acute or chronic inflammatory diseases, autoimmune disorders, active malignancies, or pre-existing anti-inflammatory therapy were excluded because these conditions could independently influence inflammatory biomarkers. Patients with incomplete clinical or laboratory data relevant to the study objectives were also excluded.

Patients were selected from the hospital’s electronic system according to the eligibility criteria, and only eligible records were exported for analysis. Therefore, the total number of patients initially screened and the number excluded for each criterion were not retained and could not be reconstructed retrospectively.

The protocol specified that, for patients with multiple admissions during the study period, only the first admission would be included. However, no patient was admitted more than once during the study interval; therefore, each patient contributed a single index admission.

### 2.2. Data Collection and Definitions

Clinical and laboratory data were extracted retrospectively from electronic medical records. Recorded variables included age, gender, BMI, HF status at admission, heart rate, duration of hospitalization, need for oxygen therapy, admission to the intensive care unit (ICU), and all-cause in-hospital mortality. Heart rate was determined from the admission electrocardiogram and confirmed on physical examination. The admission value was used in all subsequent analyses.

Laboratory data collected at admission included hemoglobin, glucose, HbA1c, lipid profile, serum iron, ferritin, NT-proBNP, sodium, potassium, albumin, and estimated glomerular filtration rate (eGFR).

Inflammatory markers included CRP, NLR, PLR, and SII. NLR was calculated by dividing the neutrophil count by the lymphocyte count; PLR, by dividing the platelet count by the lymphocyte count; and SII, by multiplying the platelet count by the neutrophil count and dividing the product by the lymphocyte count.

These parameters were selected to evaluate the relationships among inflammatory activation, metabolic abnormalities, nutritional status, and short-term outcomes in hospitalized patients with HF.

NT-proBNP was routinely measured at admission, except when reagents were temporarily unavailable.

Complete blood counts were performed using XN-1000 and XN-3000 automated hematology analyzers and manufacturer-specific reagents (Sysmex Corporation, Kobe, Japan). Routine biochemical and immunochemical assays were performed using the Cobas 8000 modular analyzer series (Roche Diagnostics GmbH, Mannheim, Germany) and an Architect system (Abbott Laboratories, Abbott Park, IL, USA), with dedicated reagents supplied by the respective manufacturers. NT-proBNP was routinely measured at admission using the PATHFAST immunoanalyzer and dedicated PATHFAST NT-proBNP reagent cartridges (PHC Corporation, Tokyo, Japan; formerly LSI Medience Corporation), except during periods of temporary reagent unavailability.

Patients were classified as having decompensated HF if, at admission, they presented with worsening HF symptoms and objective evidence of congestion (e.g., dyspnea, pulmonary rales, peripheral edema, or elevated jugular venous pressure) and required immediate intensification of HF therapy, including intravenous diuretics. Patients without evidence of acute worsening or congestion at admission were classified as having compensated HF. ICU admission was determined by the treating physicians according to clinical severity. The main indications included severe respiratory failure, persistent hypoxemia despite conventional oxygen therapy, need for ventilatory support, hemodynamic instability, need for inotropic or vasopressor support, severe pulmonary edema, or close monitoring because of rapid clinical deterioration. Treatment intensification during hospitalization was not used to reclassify patients into the decompensated HF group.

Patients were categorized as having a BMI < 25 kg/m^2^ or a BMI ≥ 25 kg/m^2^. The BMI cutoff of 25 kg/m^2^ was chosen for the primary analysis to distinguish patients with low or normal BMI from those who were overweight or obese, while preserving adequate subgroup sizes after stratification by HF status. This approach also avoided excessive subgroup fragmentation given the small number of in-hospital deaths. Because this dichotomization may oversimplify the relationship between BMI and outcome, an additional descriptive analysis was performed using standard BMI categories: underweight (<18.5 kg/m^2^), normal weight (18.5–24.9 kg/m^2^), overweight (25.0–29.9 kg/m^2^), and obesity (≥30 kg/m^2^).

Echocardiographic data, including left ventricular ejection fraction, were not available for all patients and were therefore not included in the analysis.

### 2.3. Patient Stratification and Outcomes

Patients were stratified according to BMI category and HF status into four groups: BMI < 25 kg/m^2^ with compensated HF, BMI < 25 kg/m^2^ with decompensated HF, BMI ≥ 25 kg/m^2^ with compensated HF, and BMI ≥ 25 kg/m^2^ with decompensated HF.

The primary outcome was all-cause in-hospital mortality. Cause-specific adjudication of death was not performed because the retrospective records did not contain sufficiently standardized information to assign a reliable primary cause of death for each patient. Secondary outcomes included ICU admission and the need for oxygen therapy during hospitalization. These outcomes were analyzed separately from HF status at admission and were not used to define the compensated or decompensated HF groups.

### 2.4. Statistical Analysis

Statistical analyses were performed using R version 4.3.2 (R Foundation for Statistical Computing, Vienna, Austria) with the stats, rms, pROC, ResourceSelection, ggplot2, logistf, and boot packages.

Continuous variables are expressed as mean ± standard deviation or median (interquartile range), depending on data distribution. Categorical variables are expressed as counts and percentages.

Between-group comparisons were performed using one-way analysis of variance or the Kruskal–Wallis test for continuous variables and the chi-square test or Fisher’s exact test for categorical variables, as appropriate.

A total of 27 in-hospital deaths occurred during the study period. Given the limited number of events, the multivariable models included only a small set of clinically relevant covariates selected a priori: age, gender, BMI category, HF status at admission, hemoglobin, eGFR, and blood glucose. Stepwise variable selection was not used.

The events-per-variable ratio was approximately 3.4; therefore, the adjusted models were considered exploratory rather than definitive prediction models. CRP and SII were analyzed in separate models because both markers may reflect related inflammatory pathways. Results are presented as odds ratios (ORs) with 95% confidence intervals (CIs).

To further assess the robustness of the findings in the context of the limited number of deaths, Firth’s penalized logistic regression was performed as a sensitivity analysis. Internal validation of model discrimination was performed using bootstrap resampling with 1000 resamples. Apparent and optimism-corrected area under the curve (AUC) values are reported.

NT-proBNP values showed a skewed distribution and were transformed using the natural logarithm [ln(NT-proBNP)] before inclusion in regression analyses. Sensitivity analyses involving NT-proBNP were restricted to patients with available values.

The proportion of missing NT-proBNP values was calculated. Patients with and without available NT-proBNP measurements were compared with respect to demographic characteristics, HF status, clinical outcomes, renal function, anemia, blood glucose, and inflammatory biomarkers. Continuous variables were compared using the *t*-test or Mann–Whitney U test, as appropriate, and categorical variables were compared using the chi-square test or Fisher’s exact test.

Because the proportion of missing data was low for most variables, missing values were not imputed, and complete-case analyses were used for the regression models.

Model discrimination was assessed using receiver operating characteristic (ROC) curve analysis and quantified by the AUC. Calibration was evaluated using the Hosmer–Lemeshow goodness-of-fit test. Restricted cubic spline analysis with four knots was used to explore the relationship between BMI and in-hospital mortality among patients with decompensated HF. Standard BMI categories were also examined in a supplementary descriptive analysis. Because of the small number of deaths within individual BMI categories, these categories were not used for additional adjusted regression models. Age was also examined in relation to short-term outcomes. Patients were stratified according to the median age of the cohort (<75 years and ≥75 years), and in-hospital mortality was compared between age groups using the chi-square test or Fisher’s exact test, as appropriate.

No adjustment was made for multiple comparisons; secondary and subgroup analyses were considered exploratory. All statistical tests were two-sided, and statistical significance was set at *p* < 0.05.

### 2.5. Ethical Considerations

The study was conducted in accordance with the Declaration of Helsinki. We retrospectively reviewed anonymized medical records generated during routine clinical care for patients hospitalized between January and May 2024. The study was approved by the Ethics Committee of the “St. Spiridon” County Emergency Clinical Hospital, Iași, Romania, on 11 December 2025 (approval no. 175). No intervention or additional study-specific data collection was performed. The requirement for informed consent was waived because the study used anonymized retrospective data.

## 3. Results

### 3.1. Baseline Characteristics

**Figure 1:**
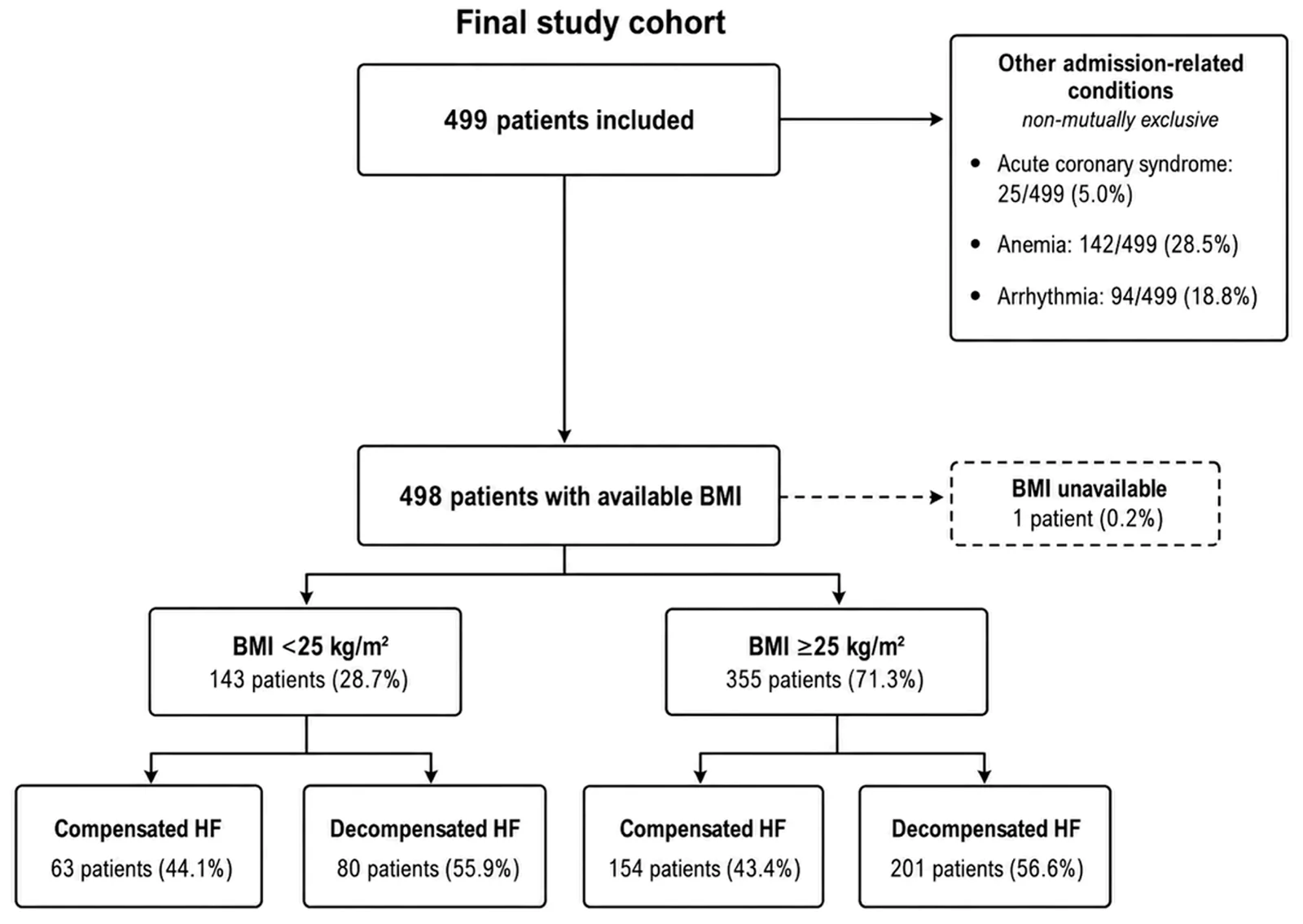
Composition and stratification of the final study cohort according to BMI and HF status. The exported database included only eligible patients. Legend: BMI—body mass index; HF—heart failure.

A total of 499 hospitalized patients with documented HF were included in the analysis. BMI was available for 498 patients; of these, 281 had decompensated HF and 217 had compensated HF at admission. These patients were divided into four BMI/HF subgroups, as shown in Figure 1.

The mean age of the study population was 74.0 ± 10.5 years. Patients with BMI < 25 kg/m^2^ were older than those with BMI ≥ 25 kg/m^2^ (*p* < 0.001).

Admission-related conditions and potential precipitating factors included acute coronary syndrome in 25 patients (5.0%), anemia in 142 (28.5%), arrhythmia in 94 (18.8%), and treatment or dietary non-adherence in 121 (24.2%). These conditions were not mutually exclusive.

Patients were further stratified according to BMI category and HF status at admission. Clinical characteristics and in-hospital outcomes are shown in Table 1, while laboratory biomarkers are listed in Table 2.

### 3.2. Clinical, Inflammatory, and Metabolic Characteristics

Patients with decompensated HF had longer hospital stays, higher heart rates, and lower hemoglobin, serum iron, albumin, and eGFR values than those with compensated HF.

Metabolic abnormalities differed across the study groups. Patients with BMI ≥ 25 kg/m^2^ generally had higher blood glucose, HbA1c, and triglyceride levels and lower high-density lipoprotein (HDL) cholesterol levels, suggesting a less favorable cardiometabolic profile. In contrast, patients with lower BMI had lower albumin and hemoglobin concentrations and higher inflammatory marker levels, a pattern consistent with a more pronounced inflammatory–catabolic state.

CRP, NLR, PLR, and SII values differed significantly across the study groups. Inflammatory marker levels were higher among patients with decompensated HF than among those with compensated HF, regardless of BMI category. The highest values were found in patients with BMI < 25 kg/m^2^ and decompensated HF.

NT-proBNP concentrations were also higher in patients with decompensated HF in both BMI categories.

ICU admission and the need for oxygen therapy were more frequent among patients with decompensated HF at admission. In-hospital mortality differed significantly among the study groups and was highest in patients with BMI < 25 kg/m^2^ and decompensated HF.

### 3.3. Clinical Outcomes

ICU admission, oxygen therapy, and in-hospital mortality differed across the four BMI/HF subgroups (*p* = 0.011, *p* < 0.001, and *p* = 0.007, respectively). The highest proportions were observed in the subgroup with BMI < 25 kg/m^2^ and decompensated HF. Mortality was highest among patients with BMI < 25 kg/m^2^ and decompensated HF and lowest among those with BMI ≥ 25 kg/m^2^ and compensated HF. Among the 498 patients with available BMI, 5 of 217 patients with compensated HF and 22 of 281 patients with decompensated HF died during hospitalization. Because causes of death were not formally adjudicated, these deaths were analyzed as all-cause in-hospital mortality.

### 3.4. BMI and Age Subgroup Analyses

When standard BMI categories were examined, in-hospital mortality was 5.0% in underweight patients, 8.9% in normal-weight patients, 3.4% in overweight patients, and 5.1% in patients with obesity (overall *p* = 0.220). Because the number of deaths within several BMI categories was small, this analysis was considered descriptive. The results are shown in Supplementary Table S2. When patients were stratified according to the median age of the cohort, in-hospital mortality was higher in patients aged ≥75 years than in those aged <75 years (7.5% vs. 3.3%, *p* = 0.039). Patients aged ≥75 years also had lower BMI values and were more likely to have BMI < 25 kg/m^2^. SII values were higher in the older age group, whereas CRP values did not differ significantly between age groups. These results are shown in Supplementary Table S3.

### 3.5. Missing NT-proBNP Data

NT-proBNP measurements were available for 405 of 499 patients (81.2%) and were missing for 94 patients (18.8%). Among the 498 patients included in the BMI- and HF-stratified analyses, measurements were available for 404 patients. NT-proBNP measurements were available for 51 of 63 patients with BMI < 25 kg/m^2^ and compensated HF, 68 of 80 patients with BMI < 25 kg/m^2^ and decompensated HF, 116 of 154 patients with BMI ≥ 25 kg/m^2^ and compensated HF, and 169 of 201 patients with BMI ≥ 25 kg/m^2^ and decompensated HF. Patients with available NT-proBNP measurements were slightly older than those without measurements (74.4 ± 10.5 vs. 72.0 ± 9.9 years, *p* = 0.036) and more frequently had decompensated HF at admission (58.8% vs. 46.8%, *p* = 0.035). ICU admission was more frequent among patients without NT-proBNP measurements (10.6% vs. 5.2%, *p* = 0.048).

No significant differences in gender, BMI, BMI category, oxygen therapy, in-hospital mortality, hemoglobin, eGFR, blood glucose, CRP, or SII were found between patients with and without NT-proBNP measurements (Supplementary Table S4).

### 3.6. Multivariable Analysis

In the CRP-based model, 471 patients were included in the complete-case analysis, including all 27 in-hospital deaths. The events-per-variable ratio was approximately 3.4. In multivariable logistic regression analysis, CRP was associated with in-hospital mortality after adjustment for selected clinical variables (adjusted OR 1.08 per 1 mg/L increase, 95% CI 1.03–1.14, *p* = 0.001). Decompensated HF was also associated with mortality (adjusted OR 3.02, 95% CI 1.04–8.78, *p* = 0.043), whereas higher eGFR values were associated with lower mortality risk (adjusted OR 0.98, 95% CI 0.96–1.00, *p* = 0.047). In the sensitivity analysis using Firth’s penalized logistic regression, CRP remained associated with in-hospital mortality (OR 1.077 per 1 mg/L increase, 95% CI 1.028–1.128, *p* = 0.0017). The complete results of the CRP-based and SII-based multivariable logistic regression models, including all covariates, are presented in Supplementary Table S5.

In the SII-based model, 485 patients were included in the complete-case analysis, including all 27 in-hospital deaths. The events-per-variable ratio was approximately 3.4. In the alternative model with SII instead of CRP, SII was associated with in-hospital mortality after adjustment for selected clinical variables (adjusted OR 1.04 per 100,000-unit increase, 95% CI 1.02–1.06, *p* < 0.001). In the sensitivity analysis using Firth’s penalized logistic regression, SII remained associated with in-hospital mortality (OR 1.041 per 100,000-unit increase, 95% CI 1.021–1.061, *p* < 0.001).

In a sensitivity analysis restricted to 389 patients with complete data, including 18 in-hospital deaths, natural-log-transformed NT-proBNP was not independently associated with in-hospital mortality after adjustment (adjusted OR 1.30 per one-unit increase in natural-log-transformed NT-proBNP, 95% CI 0.85–1.97, *p* = 0.225).

### 3.7. Model Performance and Additional Analyses

**Figure 2:**
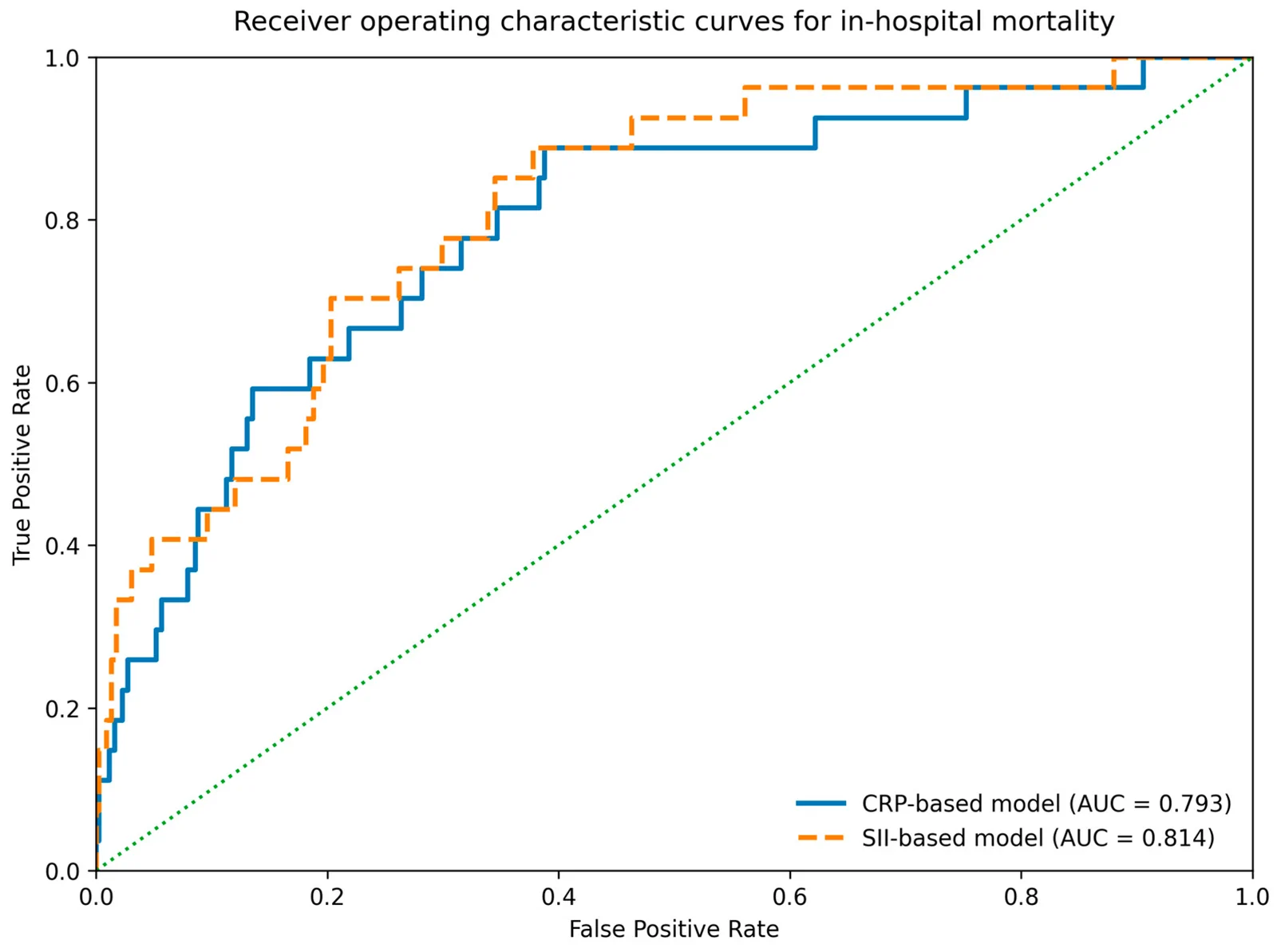
Apparent ROC curves of the multivariable logistic regression models for in-hospital mortality. Legend: CRP—C-reactive protein; AUC—area under the curve; SII—systemic immune–inflammation index. The ROC curves and AUC values shown in the figure represent apparent model discrimination. Optimism-corrected AUC values after bootstrap internal validation were 0.729 for the CRP-based model and 0.754 for the SII-based model. Because the two models were fitted in different complete-case samples, their AUC values were not formally compared.

The apparent AUC was 0.793 for the CRP-based model and 0.814 for the SII-based model, as shown in Figure 2. After bootstrap internal validation with 1000 resamples, the optimism-corrected AUC values were 0.729 and 0.754, respectively. The Hosmer–Lemeshow test showed no evidence of lack of fit for either model (*p* = 0.817 and *p* = 0.511, respectively).

The incremental value of CRP and SII was also examined by comparing the clinical models before and after inclusion of each inflammatory marker. In the CRP complete-case analysis, the AUC increased from 0.762 in the clinical model to 0.793 after CRP was added. The Akaike information criterion (AIC) decreased from 199.8 to 191.7, and the likelihood-ratio test was significant (*p* = 0.0015).

For SII, the AUC increased from 0.765 to 0.814 after the marker was added to the clinical model. The AIC decreased from 200.9 to 184.2, and the likelihood-ratio test was significant (*p* < 0.001).

Adding CRP or SII resulted in a modest improvement in model discrimination. These markers may complement the clinical variables used in the models, but the present results do not establish their clinical predictive utility. External validation is needed before either model can be considered for clinical use. The nested model comparisons are presented in Supplementary Table S6.

**Figure 3:**
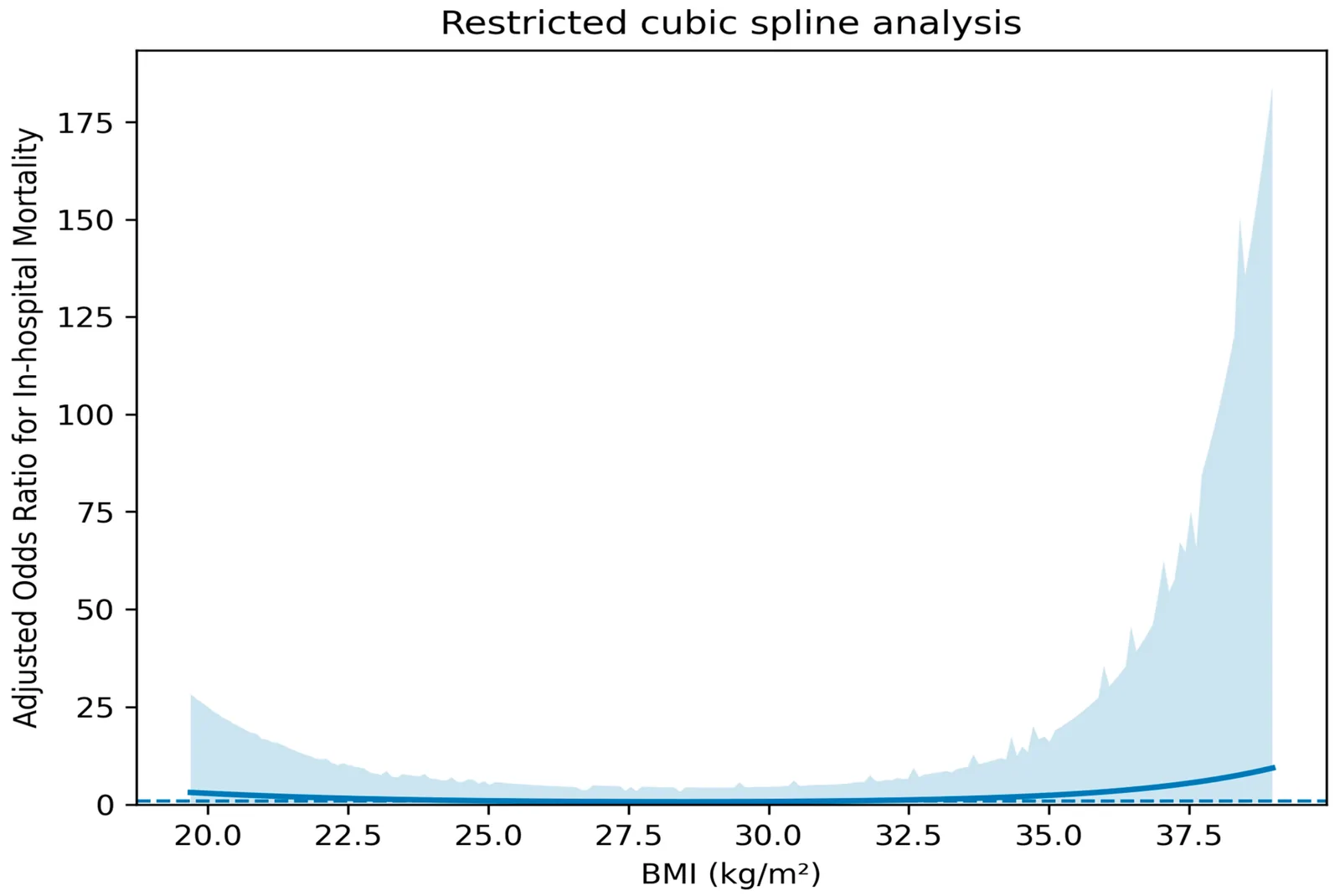
Restricted cubic spline analysis of the association between BMI and in-hospital mortality in patients with decompensated HF. Legend: BMI—body mass index; kg/m^2^—kilograms per square meter. The shaded area represents the 95% confidence interval. The spline analysis was exploratory, and the wide confidence intervals at higher BMI values indicate sparse observations and limited precision in this range.

The spline curve did not suggest a simple linear association between BMI and in-hospital mortality (Figure 3). Mortality was higher at lower BMI values and appeared lower in the intermediate BMI range. At higher BMI values, the CI became markedly wider, so the estimates in this range should be interpreted with caution. This analysis was therefore considered exploratory.

**Figure 4:**
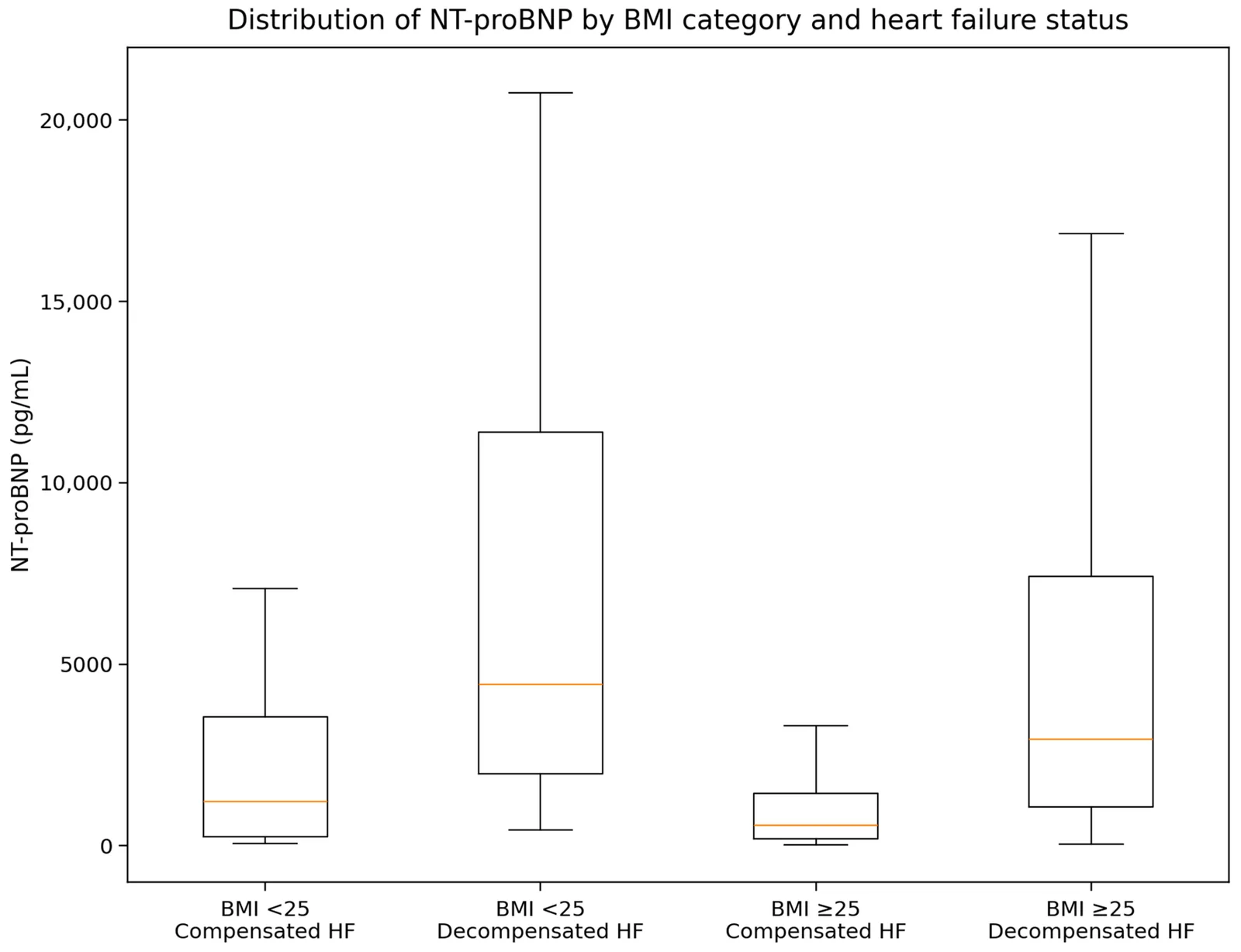
Distribution of NT-proBNP levels according to BMI category and HF status. Legend: NT-proBNP—N-terminal pro-B-type natriuretic peptide; BMI—body mass index; pg/mL—picograms per milliliter; HF—heart failure.

NT-proBNP levels varied across the four study groups and were highest in patients with decompensated HF and BMI < 25 kg/m^2^ (Figure 4).

## 4. Discussion

We conducted a retrospective single-center study of patients with established chronic HF who were admitted with a range of acute medical conditions. Some patients had decompensated HF at admission, but HF exacerbation was not the reason for hospitalization in all cases. Information on HF etiology, ejection fraction, long-term treatment, and device therapy was incomplete. These variables could not be included in the adjusted models. The mixed clinical profile of the cohort may have affected both inflammatory marker levels and in-hospital mortality and should be considered when interpreting the results.

Patients with decompensated HF at admission had higher inflammatory marker levels, and both CRP and SII remained associated with in-hospital mortality after adjustment for selected clinical variables.

Adding CRP or SII produced a modest increase in AUC and improved model fit. These findings suggest that both markers may complement routine clinical variables in short-term risk assessment. However, the lower optimism-corrected AUC values and the limited number of deaths warrant cautious interpretation. The models remain exploratory and require external validation before either can be considered for clinical use.

Inflammatory marker levels, metabolic abnormalities, and nutritional impairment were frequently observed in hospitalized patients with HF, particularly during decompensation. These parameters may therefore help assess disease severity during hospitalization.

Chronic inflammatory activation has increasingly been linked to several processes involved in HF progression, including endothelial dysfunction, altered glucose metabolism, skeletal muscle loss, and progressive nutritional impairment. Metabolic abnormalities are also common in older patients with multiple comorbidities and may contribute to the inflammatory profile observed during hospitalization [32,33].

CRP, NLR, PLR, and SII were higher in decompensated HF than in compensated HF in both BMI groups. Patients with decompensated HF and BMI < 25 kg/m^2^ had the highest values. CRP and SII remained associated with mortality after adjustment. However, these associations do not establish the clinical predictive value of either marker. Although patients with recognized infections or inflammatory diseases were excluded, CRP and SII remain nonspecific markers. Their levels may also reflect occult infection, ischemia, renal dysfunction, congestion, tissue injury, or other acute conditions. These findings should therefore not be interpreted as evidence of HF-specific inflammatory activation. The sensitivity analyses yielded similar results with penalized regression.

Previous studies evaluating inflammatory biomarkers in HF have similarly reported associations between elevated CRP levels and adverse short-term outcomes, although differences in study design, patient populations, and clinical endpoints limit direct comparison [34,35]. Similar associations have been described for hematologic inflammatory indices such as PLR, NLR, and SII across both chronic and acute cardiovascular conditions [36,37]. However, many previous studies evaluated isolated biomarkers or selected patient populations without simultaneous assessment of BMI category, metabolic abnormalities, and short-term in-hospital outcomes [34,38,39].

Metabolic differences were observed between BMI groups. Patients with a BMI ≥ 25 kg/m^2^ generally had higher blood glucose, HbA1c, and triglyceride levels and lower HDL cholesterol levels. Patients with lower BMI more frequently had anemia, lower albumin levels, and higher inflammatory marker levels at admission.

When patients were dichotomized using a BMI cutoff of 25 kg/m^2^, in-hospital mortality was numerically higher in the BMI < 25 kg/m^2^ group, but the difference was not statistically significant. The analysis based on standard BMI categories also showed no significant overall difference in mortality. These findings should therefore not be considered definitive evidence of an obesity paradox. Mortality was numerically lower among overweight patients, whereas estimates for the underweight and obesity groups were imprecise because of the small number of deaths. BMI should be regarded mainly as a broad marker of nutritional and metabolic status because it does not distinguish between adiposity, muscle mass, nutritional status, and fluid overload in hospitalized patients with HF [40,41]. Age may partly explain the numerical difference in mortality between the BMI groups. In this cohort, patients with lower BMI were older, and patients aged ≥75 years had higher crude in-hospital mortality and higher SII values. Although age was included in the adjusted regression models and was not independently associated with mortality, lower BMI should be interpreted as part of a broader frailty-related and inflammatory phenotype rather than as an isolated causal factor.

The findings from the restricted cubic spline analysis suggested a possible nonlinear association between BMI and in-hospital mortality. However, this finding should be interpreted cautiously because the CIs widened at higher BMI values, where observations were sparse. The spline curve was therefore considered exploratory and was not used to define a specific risk pattern at high BMI levels.

Other possible explanations include cardiac cachexia, frailty, sarcopenia, reverse causality, and residual confounding. Cachexia, frailty, and sarcopenia are more common in patients with advanced HF and lower BMI. Thus, our results should not be taken to imply a causal protective effect of obesity.

Some studies have reported lower mortality among overweight and obese hospitalized patients with HF, whereas others have suggested that this association may be influenced by age, frailty, nutritional status, or disease severity [40,42]. In the present cohort, mortality was numerically higher in patients with BMI < 25 kg/m^2^, although the difference was not statistically significant; this finding should not be interpreted as evidence that obesity itself is protective. Furthermore, waist circumference, a measure of central adiposity not captured by BMI, was not available in this retrospective analysis. Future studies should include waist circumference and, where possible, other measures of body composition. These data may help clarify whether metabolic risk in HF is related to BMI itself or rather to body fat distribution.

NT-proBNP showed a different pattern from the inflammatory markers. Although concentrations were higher in patients with decompensated HF, NT-proBNP was not independently associated with mortality after adjustment in this cohort. Missing NT-proBNP values, the relatively small number of outcome events, and overlap with other markers of disease severity may have contributed to these findings.

NT-proBNP and inflammatory markers likely reflect different aspects of HF severity. NT-proBNP primarily reflects myocardial wall stress and congestion, whereas inflammatory marker levels may also be affected by renal dysfunction, infection, tissue hypoperfusion, or other conditions present during hospitalization [43,44,45]. These differing patterns may partly reflect the clinical heterogeneity of the hospitalized HF population.

In routine clinical practice, inflammatory and metabolic parameters are often available at hospital admission and do not require specialized testing. Although these markers should not be interpreted in isolation, they may help assess short-term clinical severity when considered together.

A strength of this study is the integrated assessment of routinely available inflammatory and metabolic variables in a real-world cohort of hospitalized patients.

Several limitations should be considered. This was a single-center retrospective analysis with only 27 in-hospital deaths, resulting in a low events-per-variable ratio and limited stability of the multivariable estimates. Although the covariates were selected a priori and CRP and SII were evaluated in separate models, residual model instability cannot be excluded despite Firth penalization and bootstrap validation. The models should therefore be considered exploratory and hypothesis-generating; they require external validation in larger multicenter cohorts.

Echocardiographic data, including left ventricular ejection fraction, were not available for all patients. We therefore could not classify the cohort as having heart failure with reduced ejection fraction (HFrEF), mildly reduced ejection fraction (HFmrEF), or preserved ejection fraction (HFpEF), nor could we perform phenotype-specific analyses. This is an important limitation because these phenotypes differ in their inflammatory profiles, metabolic characteristics, comorbidities, treatment, and prognosis. The associations reported in this study should not be assumed to apply equally to all HF phenotypes. Studies with systematic echocardiographic assessment are needed to examine these differences.

In addition, detailed data on HF etiology, precipitating causes of decompensation, background pharmacological therapy, device therapy (including cardiac resynchronization therapy), and electrocardiographic characteristics were incomplete. These factors may influence inflammatory biomarker levels and clinical outcomes but could not be included in the adjusted analyses. This is an important limitation of the study. Future prospective studies should systematically collect these data and examine their potential effects on inflammatory biomarkers and prognosis.

The cohort included patients with chronic HF admitted for different acute medical conditions, and HF exacerbation was not always the reason for admission. This clinical heterogeneity may have influenced inflammatory marker levels and all-cause in-hospital mortality. Therefore, the findings should not be considered specific to patients admitted for acute HF.

The retrospective database did not include a separate screening log or a standardized, mutually exclusive primary discharge diagnosis. Consequently, the number of exclusions for each criterion could not be reconstructed, and admissions could not be reliably classified as primarily cardiac or noncardiac.

Data on noninvasive ventilation (NIV) and its timing relative to ICU admission were not systematically available. Therefore, NIV use could not be analyzed as a separate treatment variable or outcome.

Although patients with identified infections, inflammatory diseases, autoimmune disorders, active malignancies, or pre-existing anti-inflammatory therapy were excluded, residual confounding remains possible. CRP and SII are nonspecific and may still be influenced by occult infection, ischemia, renal dysfunction, congestion, tissue injury, or other acute medical conditions.

The exact interval between the first diagnosis of HF and the index hospitalization was not systematically available in the medical records. Therefore, disease duration could not be included in the analysis. This may be relevant because patients with recently diagnosed HF and those with long-standing HF may differ in clinical profile, inflammatory status, treatment exposure, and prognosis.

BMI was used as a simple anthropometric measure, but it does not adequately reflect body composition, visceral adiposity, sarcopenia, frailty, or nutritional reserve. Direct measures of frailty and muscle mass were not available.

Inflammatory markers were evaluated only at admission, and serial measurements during hospitalization were unavailable. NT-proBNP values were unavailable in 94 patients (18.8%), which limited the NT-proBNP sensitivity analyses. Although patients with and without available NT-proBNP measurements did not differ significantly with respect to in-hospital mortality, BMI, renal function, CRP, or SII, some differences were observed in age, HF decompensation status, and ICU admission. Therefore, selection bias related to NT-proBNP testing cannot be completely excluded. The primary causes of in-hospital death were not formally adjudicated; accordingly, mortality was analyzed as all-cause in-hospital mortality, and cause-specific mortality patterns, including deaths among patients classified as compensated at admission, could not be examined in detail. In addition, only in-hospital outcomes were evaluated, without information regarding mortality or rehospitalization after discharge.

## 5. Conclusions

Higher CRP and SII levels were associated with all-cause in-hospital mortality in exploratory adjusted models, and these associations remained consistent in penalized sensitivity analyses. Mortality was numerically, but not significantly, higher in patients with BMI < 25 kg/m^2^.

Patients with a higher BMI had less favorable glycemic and lipid profiles, whereas those with a lower BMI showed a more pronounced inflammatory–catabolic pattern. This finding requires cautious interpretation because BMI does not distinguish among adiposity, fluid retention, nutritional status, frailty, and muscle mass, all of which may be relevant in hospitalized patients with HF.

NT-proBNP concentrations were higher in patients with decompensated HF at admission but were not independently associated with mortality after adjustment in this cohort. Routinely available inflammatory and metabolic parameters may provide complementary information during clinical assessment, but their role in risk stratification requires confirmation in external cohorts.

## Figures and Tables

**Table 1:** Clinical characteristics, routine laboratory findings, and hospital outcomes across BMI and HF subgroups.

Variable	BMI < 25 and Compensated HF (*n* = 63)	BMI < 25 and Decompensated HF (*n* = 80)	BMI ≥ 25 and Compensated HF (*n* = 154)	BMI ≥ 25 and Decompensated HF (*n* = 201)	*p*-Value
Age, years	77.1 ± 7.2	78.8 ± 9.9	70.6 ± 10.3	73.6 ± 10.7	<0.001
Length of stay, days	6.8 ± 3.8	7.6 ± 3.4	6.3 ± 3.9	7.8 ± 3.9	0.003
Heart rate, bpm	81.5 ± 22.1	89.7 ± 23.1	81.1 ± 16.6	89.3 ± 22.6	<0.001
Hemoglobin, g/dL	12.5 ± 3.0	11.7 ± 2.4	13.4 ± 2.6	12.4 ± 2.5	<0.001
Serum iron, µg/dL	70.0 (45.0–96.0)	41.0 (22.0–56.0)	73.0 (46.2–99.5)	48.0 (31.0–74.0)	<0.001
Sodium, mmol/L	139.6 ± 3.5	138.5 ± 4.9	139.4 ± 4.7	139.1 ± 4.2	0.371
Potassium, mmol/L	4.3 ± 0.7	4.4 ± 0.6	4.5 ± 0.6	4.5 ± 0.6	0.281
eGFR, mL/min/ 1.73 m^2^	69.6 ± 23.1	63.2 ± 23.1	73.8 ± 22.5	65.0 ± 24.4	0.001
Albumin, g/dL	4.0 ± 0.6	3.4 ± 0.8	3.9 ± 0.5	3.8 ± 0.5	<0.001
ICU admission, *n* (%)	2 (3.2%)	11 (13.8%)	5 (3.2%)	13 (6.5%)	0.011
Oxygen therapy, *n* (%)	10 (15.9%)	38 (47.5%)	12 (7.8%)	79 (39.3%)	<0.001
In-hospital mortality, *n* (%)	2 (3.2%)	10 (12.5%)	3 (1.9%)	12 (6.0%)	0.007

Legend: Continuous variables are presented as mean ± standard deviation or median (interquartile range), as appropriate. Categorical variables are presented as counts and percentages. BMI—body mass index; HF—heart failure; bpm—beats per minute; eGFR—estimated glomerular filtration rate; ICU—intensive care unit. The reported *p*-values refer to the global comparison across the four BMI/HF subgroups. Pairwise comparisons between the two decompensated HF groups stratified by BMI are presented in Supplementary Table S1.

**Table 2:** Metabolic, cardiac, and inflammatory biomarkers across BMI and HF subgroups.

Variable	BMI < 25 and Compensated HF (*n* = 63)	BMI < 25 and Decompensated HF (*n* = 80)	BMI ≥ 25 and Compensated HF (*n* = 154)	BMI ≥ 25 and Decompensated HF (*n* = 201)	*p*-Value
Glucose, mg/dL	104.0 ± 20.2	121.7 ± 46.4	119.7 ± 51.5	125.3 ± 46.9	0.019
HbA1c, %	5.8 ± 0.6	6.1 ± 1.1	6.4 ± 1.4	6.6 ± 1.4	0.003
Total cholesterol, mg/dL	166.6 ± 51.5	143.5 ± 39.7	168.3 ± 52.3	145.3 ± 46.0	<0.001
LDL cholesterol, mg/dL	102.0 ± 43.0	86.1 ± 31.5	102.6 ± 44.7	87.4 ± 38.0	<0.001
HDL cholesterol, mg/dL	47.7 ± 17.7	38.4 ± 14.8	42.6 ± 12.9	38.3 ± 14.2	<0.001
Triglycerides, mg/dL	75.5 (63.8–100.5)	80.0 (61.0–100.0)	103.5 (79.0–134.0)	82.0 (64.0–110.0)	<0.001
Ferritin, ng/mL	140.5 (74.5–213.8)	98.0 (39.0–207.5)	87.5 (57.2–154.2)	82.0 (45.0–139.0)	0.029
NT-proBNP, pg/mL	1219 (251–3548), n = 51	4447.5 (1986.5–11,405.5), n = 68	562 (202.5–1450), n = 116	2935 (1072.5–7420.8), n = 169	<0.001
CRP, mg/L	0.4 (0.1–2.0)	1.0 (0.4–2.4)	0.4 (0.2–1.4)	1.0 (0.4–2.8)	<0.001
PLR	143.5 (93.8–198.8)	200.2 (116.6–274.9)	136.2 (101.5–188.3)	162.8 (113.8–222.0)	<0.001
NLR	3.4 (2.2–4.9)	4.8 (3.3–7.7)	2.8 (1.9–4.0)	3.8 (2.5–6.3)	<0.001
SII (×10^3^)	701.4 (296.5–1055.4)	1001.0 (589.4–1694.0)	651.5 (445.4–1025.4)	808.7 (501.3–1594.0)	<0.001

Legend: Continuous variables are presented as mean ± standard deviation or median (interquartile range), as appropriate. BMI—body mass index; HF—heart failure; HbA1c—glycated hemoglobin; LDL—low-density lipoprotein; HDL—high-density lipoprotein; NT-proBNP—N-terminal pro-B-type natriuretic peptide; CRP—C-reactive protein; NLR—neutrophil-to-lymphocyte ratio; PLR—platelet-to-lymphocyte ratio; SII—systemic immune–inflammation index. NT-proBNP values are reported only for patients with available measurements, as indicated in the corresponding table cells. The reported *p*-values refer to the global comparison across the four BMI/HF subgroups. Pairwise comparisons between the two decompensated HF groups stratified by BMI are shown in Supplementary Table S1.

## Data Availability

The original contributions presented in this study are included in the article and Supplementary Material. Further inquiries can be directed to the corresponding author.

## Supplementary Materials

The following supporting information can be downloaded at: https://www.mdpi.com/article/10.3390/metabo16080534/s1, Table S1: Pairwise comparison between decompensated HF patients stratified by BMI; Table S2: In-hospital mortality according to standard BMI categories; Table S3: Clinical outcomes and inflammatory markers according to median age; Table S4: Comparison between patients with and without available NT-proBNP measurements; Table S5: Complete multivariable logistic regression models for in-hospital mortality; Table S6: Comparison of clinical base models and nested models including inflammatory biomarkers.

## Abbreviations

The following abbreviations are used in this manuscript:

AIC	Akaike information criterion
AUC	Area under the curve
BMI	Body mass index
CI	Confidence interval
CRP	C-reactive protein
eGFR	Estimated glomerular filtration rate
ESC	European Society of Cardiology
HbA1c	Glycated hemoglobin
HDL	High-density lipoprotein
HF	Heart failure
ICU	Intensive care unit
LDL	Low-density lipoprotein
NIV	Non-invasive ventilation
NLR	Neutrophil-to-lymphocyte ratio
NT-proBNP	N-terminal pro-B-type natriuretic peptide
OR	Odds ratio
PLR	Platelet-to-lymphocyte ratio
ROC	Receiver operating characteristic
SII	Systemic immune–inflammation index
