# Supplementary material for: Inflammatory and Metabolic Biomarkers Associated with In-Hospital Mortality in Patients Hospitalized with Heart Failure
Source: Metabolites. 2026 Jul 29;16(8):534. doi: 10.3390/metabo16080534 (PMC13515100; doi:10.3390/metabo16080534)
Supplement: Supplementary file 1 [file metabolites-16-00534-s001.zip › Supplementary Table S1.pdf]

Supplementary Table S1. Pairwise comparison between decompensated HF patients stratified by BMI.

Variable	BMI <25 kg/m² and decompensated HF (*n*=80)	BMI ≥25 kg/m² and decompensated HF (*n*=201)	p-value
Age, years	78.8 ± 9.9	73.6 ± 10.7	< 0.001
Length of stay, days	7.6 ± 3.4	7.8 ± 3.9	0.738
Heart rate, bpm	89.7 ± 23.1	89.4 ± 22.6	0.930
Hemoglobin, g/dL	11.7 ± 2.4	12.4 ± 3.0	0.024
Serum iron, µg/dL	41.0 (22.0–56.0)	48.0 (31.0–73.8)	0.038
Sodium, mmol/L	138.5 ± 4.9	139.6 ± 3.5	0.331
Potassium, mmol/L	4.4 ± 0.6	4.3 ± 0.7	0.204
eGFR, mL/min/ 1.73 m²	63.2 ± 23.1	65.0 ± 24.4	0.558
Albumin, g/dL	3.4 ± 0.8	4.0 ± 0.6	0.006
ICU admission, *n* (%)	11 (13.8%)	13 (6.5%)	0.059
Oxygen therapy, *n* (%)	38 (47.5%)	79 (39.3%)	0.229
In-hospital mortality, *n* (%)	10 (12.5%)	12 (6.0%)	0.084
Glucose, mg/dL	121.7 ± 46.4	125.3 ± 47.0	0.567
HbA1c, %	6.1 ± 1.1	6.6 ± 1.4	0.019
Total cholesterol, mg/dL	143.5 ± 39.7	145.3 ± 46.0	0.766
LDL cholesterol, mg/dL	86.1 ± 31.5	87.4 ± 38.0	0.779
HDL cholesterol, mg/dL	38.4 ± 14.8	38.3 ± 14.2	0.979

Triglycerides, mg/dL	80.0 (61.0–100.0)	82.0 (64.0–110.0)	0.190
Ferritin, ng/mL	98.0 (39.0–207.5)	82.0 (45.0–138.0)	0.302
NT-proBNP, pg/mL	4447.5 (1986.5–11405.5)	2935.0 (1072.5–7420.8)	0.008
CRP, mg/L	1.0 (0.4–2.4)	1.0 (0.4–2.8)	0.643
PLR	200.2 (116.6–274.9)	162.7 (113.7–221.6)	0.062
NLR	4.8 (3.3 – 7.7)	3.8 (2.5 – 6.3)	0.017
SII ($\times 10^3$)	1001.0 (589.5–1694.0)	808.7 (501.3–1594.0)	0.116

Legend: BMI - body mass index; HF - heart failure; eGFR - estimated glomerular filtration rate; ICU - intensive care unit; HbA1c - hemoglobin A1c; LDL - low-density lipoprotein; HDL - high-density lipoprotein; NT-proBNP - N-terminal pro-B-type natriuretic peptide; CRP - C-reactive protein; PLR - platelet-to-lymphocyte ratio; NLR - neutrophil-to-lymphocyte ratio; SII - systemic immune-inflammation index.
