# Supplementary material for: Inflammatory and Metabolic Biomarkers Associated with In-Hospital Mortality in Patients Hospitalized with Heart Failure
Source: Metabolites. 2026 Jul 29;16(8):534. doi: 10.3390/metabo16080534 (PMC13515100; doi:10.3390/metabo16080534)
Supplement: Supplementary file 1 [file metabolites-16-00534-s001.zip › Supplementary Table S2.pdf]

Supplementary Table S2. In-hospital mortality according to standard BMI categories.

**BMI category**	***n***	**In-hospital deaths, *n* (%)**
Underweight, <18.5 kg/m²	20	1 (5.0%)
Normal weight, 18.5–24.9 kg/m²	124	11 (8.9%)
Overweight, 25.0–29.9 kg/m²	179	6 (3.4%)
Obesity, ≥ 30.0 kg/m²	176	9 (5.1%)

* Legend: BMI - body mass index. The p-value for comparison across standard BMI categories was 0.220.
