# Supplementary material for: Inflammatory and Metabolic Biomarkers Associated with In-Hospital Mortality in Patients Hospitalized with Heart Failure
Source: Metabolites. 2026 Jul 29;16(8):534. doi: 10.3390/metabo16080534 (PMC13515100; doi:10.3390/metabo16080534)
Supplement: Supplementary file 1 [file metabolites-16-00534-s001.zip › Supplementary Table S3.pdf]

Supplementary Table S3. Clinical outcomes and inflammatory markers according to median age.

Variable	Age <75 years (n=244)	Age ≥75 years (n=255)	*p*-value
In-hospital mortality, *n* (%)	8 (3.3%)	19 (7.5%)	0.039
BMI, kg/m²	30.1 ± 6.8	27.1 ± 5.7	<0.001
BMI <25 kg/m², *n* (%)	49 (20.1%)	94 (37.0%)	<0.001
Decompensated HF, *n* (%)	124 (50.8%)	158 (62.0%)	0.012
CRP, mg/L	0.68 (0.24–2.30)	0.83 (0.26–2.43)	0.504
SII (×10³)	674.8 (425.0–1311.9)	791.8 (577.9–1457.6)	0.003

Legend: BMI - body mass index; HF - heart failure; CRP - C-reactive protein; SII - systemic immune-inflammation index.
