# Supplementary material for: Inflammatory and Metabolic Biomarkers Associated with In-Hospital Mortality in Patients Hospitalized with Heart Failure
Source: Metabolites. 2026 Jul 29;16(8):534. doi: 10.3390/metabo16080534 (PMC13515100; doi:10.3390/metabo16080534)
Supplement: Supplementary file 1 [file metabolites-16-00534-s001.zip › Supplementary Table S4.pdf]

Supplementary Table S4. Comparison between patients with and without available NT-proBNP measurements.

Variable	NT-proBNP available (*n*=405)	NT-proBNP missing (*n*=94)	*p*-value
Age, years	74.4 ± 10.5	72.0 ± 9.9	0.036
Male sex, *n* (%)	201 (49.6)	52 (55.3)	0.320
BMI, kg/m²	28.3 ± 6.5	29.5 ± 6.2	0.102
BMI <25 kg/m², *n* (%)	120 (29.6)	24 (25.5)	0.430
Decompensated HF, *n* (%)	238 (58.8)	44 (46.8)	0.035
ICU admission, *n* (%)	21 (5.2)	10 (10.6)	0.048
Oxygen therapy, *n* (%)	114 (28.1)	26 (27.7)	0.924
In-hospital mortality, *n* (%)	21 (5.2)	6 (6.4)	0.644
Hemoglobin, g/dL	12.6 ± 2.6	12.7 ± 3.0	0.748
eGFR, mL/min/1.73 m²	67.7 ± 23.6	69.7 ± 24.6	0.461
Glycemia, mg/dL	121.5 ± 47.0	114.5 ± 42.4	0.169
CRP, mg/L	0.8 (0.3–2.4)	0.5 (0.2–2.3)	0.151
SII (×10³)	755.4 (492.3–1420.1)	712.2 (433.7–1100.2)	0.246

* Legend: Continuous variables are presented as mean ± standard deviation or median (interquartile range), as appropriate. Categorical variables are presented as counts and percentages. BMI - body mass index; HF - heart failure; ICU - intensive care unit; eGFR - estimated glomerular filtration rate; CRP - C-reactive protein; SII - systemic immune-inflammation index.
