# Supplementary material for: Inflammatory and Metabolic Biomarkers Associated with In-Hospital Mortality in Patients Hospitalized with Heart Failure
Source: Metabolites. 2026 Jul 29;16(8):534. doi: 10.3390/metabo16080534 (PMC13515100; doi:10.3390/metabo16080534)
Supplement: Supplementary file 1 [file metabolites-16-00534-s001.zip › Supplementary Table S5.pdf]

Supplementary Table S5. Complete multivariable logistic regression models for in-hospital mortality.

Variable	CRP-based model OR (95% CI)	*p*-value	SII-based model OR (95% CI)	*p*-value
Age, per year	1.02 (0.97–1.07)	0.385	1.01 (0.96–1.06)	0.726
Male sex	1.82 (0.73–4.53)	0.199	2.01 (0.79–5.13)	0.142
BMI <25 kg/m^2	1.58 (0.65–3.81)	0.312	2.06 (0.84–5.02)	0.113
Decompensated HF	3.00 (1.03–8.73)	0.044	2.86 (0.95–8.60)	0.062
Hemoglobin, per 1 g/dL	0.95 (0.80–1.12)	0.533	0.96 (0.81–1.13)	0.601
eGFR, per 1 mL/min/ 1.73 m^2	0.98 (0.96–1.00)	0.045	0.98 (0.96–1.00)	0.065
Glycemia, per 1 mg/dL	1.00 (0.99–1.01)	0.944	1.00 (0.99–1.01)	0.587
CRP, per 1 mg/L	1.08 (1.03–1.14)	0.001	—	—
SII, per 100,000-unit increase	—	—	1.04 (1.02–1.06)	< 0.001

Legend: BMI - body mass index; HF - heart failure; eGFR - estimated glomerular filtration rate; CRP - C-reactive protein; SII - systemic immune-inflammation index; OR - odds ratio; CI - confidence interval. The CRP-based model included 472 patients and 27 in-hospital deaths. The SII-based model included 486 patients and 27 in-hospital deaths.
