# Supplementary material for: Inflammatory and Metabolic Biomarkers Associated with In-Hospital Mortality in Patients Hospitalized with Heart Failure
Source: Metabolites. 2026 Jul 29;16(8):534. doi: 10.3390/metabo16080534 (PMC13515100; doi:10.3390/metabo16080534)
Supplement: Supplementary file 1 [file metabolites-16-00534-s001.zip › Supplementary Table S6.pdf]

Supplementary Table S6. Comparison of clinical base models and nested models including inflammatory biomarkers.

Model comparison	*n*	Deaths	AUC base model	AUC extended model	Change in AUC	AIC base model	AIC extended model	*p*-value
Clinical model *vs*. clinical model + CRP	472	27	0.762	0.793	0.031	199.8	191.7	0.0015
Clinical model *vs*. clinical model + SII	486	27	0.765	0.813	0.049	200.9	184.2	<0.001

Legend: CRP - C-reactive protein; SII - systemic immune-inflammation index; AUC - area under the curve; AIC - Akaike information criterion.
